# Immunogenic *Mycobacterium africanum* Strains Associated with Ongoing Transmission in The Gambia

**DOI:** 10.3201/eid1910.121023

**Published:** 2013-10

**Authors:** Florian Gehre, Martin Antonio, Jacob K. Otu, Neneh Sallah, Oumie Secka, Tutty Faal, Patrick Owiafe, Jayne S. Sutherland, Ifedayo M. Adetifa, Martin O. Ota, Beate Kampmann, Tumani Corrah, Bouke C. de Jong

**Affiliations:** Medical Research Council Unit, Fajara, The Gambia (F. Gehre, M. Antonio, J.K. Otu, N. Sallah, O. Secka, T. Faal, P. Owiafe, J.S. Sutherland, I.M. Adetifa, M.O. Ota, B. Kampmann, T. Corrah, B.C. de Jong);; Institute for Tropical Medicine, Antwerp, Belgium (F. Gehre, B.C. de Jong);; Imperial College London, London, UK (B. Kampmann);; New York University, New York, New York, USA (B.C. de Jong)

**Keywords:** Mycobacterium tuberculosis, Mycobacterium africanum, spoligotyping, population structure, transmission, ELISPOT, tuberculosis and other mycobacteria, immunogenicity, human hosts, The Gambia, shared international type, SIT, PPD

## Abstract

In West Africa, *Mycobacterium tuberculosis* strains co-circulate with *M. africanum*, and both pathogens cause pulmonary tuberculosis in humans. Given recent findings that *M. tuberculosis* T-cell epitopes are hyperconserved, we hypothesized that more immunogenic strains have increased capacity to spread within the human host population. We investigated the relationship between the composition of the mycobacterial population in The Gambia, as measured by spoligotype analysis, and the immunogenicity of these strains as measured by purified protein derivative–induced interferon-γ release in ELISPOT assays of peripheral blood mononuclear cells. We found a positive correlation between strains with superior spreading capacity and their relative immunogenicity. Although our observation is true for *M. tuberculosis* and *M. africanum* strains, the association was especially pronounced in 1 *M. africanum* sublineage, characterized by spoligotype shared international type 181, which is responsible for 20% of all tuberculosis cases in the region and therefore poses a major public health threat in The Gambia.

Tuberculosis (TB), caused by bacterial pathogens of the *Mycobacterium tuberculosis* complex (MTBC), is a major global health problem. Sub-Saharan Africa has the highest rate of TB per capita and the lowest case detection rate; although TB incidence is decreasing globally, incidence rates are increasing in most countries in the West Africa region (*1*). Moreover, almost half of all TB cases in West Africa are caused by infection with an unusual member of the MTBC, *M. africanum*, a lineage found exclusively in this region. Although *M. africanum* was initially described in Senegal in 1968 (*2*), and despite its importance and high prevalence in this region, relatively little is known about the bacterium (*3*). In general, *M. africanum* can be divided into 2 lineages: Afri_1, by SpolDB4 definition (*4*), corresponding to the green lineage 6 (*5*), which has the highest prevalence in Senegal, Mali, The Gambia, Guinea-Bissau, and Sierra Leone (*3*); and Afri_2 (*4*), corresponding to the brown lineage 5 (*5*), which is mainly found in the eastern part of West Africa, in countries such as Côte d’Ivoire, Ghana, Benin, Nigeria, and Cameroon (*3*).

Although transmission of *M. africanum* from host to host is a crucial element of the spread of the disease, the underlying biological mechanisms triggering transmission are elusive. We assessed transmission dynamics and interaction between the 2 mycobacterial populations in The Gambia, a country in western West Africa, and compared the local situation with previously published data from Guinea-Bissau, another country within the region (*6*). In particular, considering a recent publication suggesting that conserved mycobacterial T-cell epitopes may play a role in the transmission of the mycobacteria within the host population (*7*), we investigated whether differences in immunogenicity between *M. tuberculosis* and *M. africanum* strains (especially of the predominant Euro-American [EA] and Afri_1 lineages) could predict the success of certain sublineages to transmit and establish themselves within the human host population.

## Materials and Methods

### Study Population and Sample Collection

Data for our study came from an ongoing TB case–contact study at the Medical Research Council in The Gambia; TB case-patients were recruited for that study during June 20, 2002–December 21, 2009. Consecutive patients were included after written informed consent if they were >15 years old, resided in the study area (Greater Banjul area), and produced 2 sputum samples that were positive for acid-fast bacilli by Ziehl-Neelsen staining.

### Spoligotyping and Analysis

Genomic DNA was purified from the collected sputum samples by using the cetyl trimethyl ammonium bromide and chloroform method, as described (*8*). Spoligotyping was performed by using commercially available membranes (Ocimum Biolsolutions, Hyderabad, India), according to standardized protocols (*9*). Spoligotype patterns with ambiguous signature were confirmed by using long-sequence polymorphism PCR.

In addition to analyzing the samples collected in The Gambia, we reanalyzed spoligotypes from published studies from Guinea-Bissau (*6*). The shared international type (SIT) number was assigned by using the SITVIT database on the Institute Pasteur de Guadeloupe website (www.pasteur-guadeloupe.fr/tb/bd_myco.html). Lineages of spoligotypes were assigned according to SpolDB4 classification by using the TB Lineage online platform (http://tbinsight.cs.rpi.edu/about_tb_lineage.html) (*10*). Spoligotype data were further analyzed by using spolTools (www.emi.unsw.edu.au/spolTools) (*11*), which provides online tools for the construction of Spoligoforests (*12*) and to Detect Emerging Strains of Tuberculosis by Using Spoligotyping (DESTUS) (*13*) from spoligotype data. As recommended by the provider, the Spoligoforests and DESTUS programs were run with the default settings.

To analyze temporal clustering of spoligotypes from The Gambia, we used SaTScan version 9.1.1 software (www.satscan.org) (*14*); we conducted a purely temporal analysis for high rates of clustering in a discrete Poisson model. Hospital admission dates for each patient were used as input dates for each spoligotype, and the resolution of the analysis was set to days.

### PPD-ELISPOT

Purified protein derivative (PPD) ELISPOT assays were performed as described (*15*) on a subset of 372 study samples. Quantitative results were expressed as the number of spot-forming units (sfu) that produce interferon-γ in response to *M. tuberculosis* PPD antigen. Positive wells were predefined as containing >10 sfu more than, and at least 2 times as many as, negative control wells. The negative control well was required to have <30 sfu.

### Statistical Analysis

Odds ratios (ORs) and 95% CIs were calculated for analysis of cross-tabulations. To estimate differences between groups, the χ^2^ test was applied with 2-tailed p values. To confirm the results and to use a more accurate test for 2 × 2 tables, we also performed Fisher exact testing. We considered test results with p values <0.05 to be statistically significant.

The recent transmission index (RTI_n–1_) was calculated as described (*16*). Patients with singleton strains were considered to have TB from reactivation and not recent transmission and, therefore, RTI_n–1_ = 0. The average PPD response of patients with singleton strains was considered the baseline. Following calculation of the PPD response of the singleton strains, all recently transmitted strains with a cluster size of 2 were included, and the average PPD response and RTI_n–1_ was re-calculated. This procedure was continued by stepwise inclusion of the next bigger genotypic cluster (i.e., [singleton] + [cluster n = 2] + [cluster n = 3]; [singleton] + [cluster n = 2] + [cluster n = 3] + [cluster n = 4]; ...), and recalculation of the average PPD response for each respective RTI_n–1_ group was performed.

## Results

### Population Structure of MTBC and Transmission of Isolates

**Figure 1:**
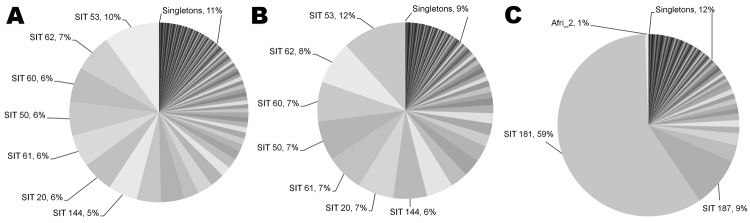
Spoligotyping results showing population structure of *Mycobacterium tuberculosis* and *M. africanum*, The Gambia, 2002–2009. A) All *M. tuberculosis* sensu stricto lineages (including Euro-American); B) Euro-American lineage; C) *M. africanum* lineages (Afri_1 and Afri_2). SIT, shared international type.

For the study period, 1,003 smear-positive TB cases were identified. Spoligotypes could be obtained from 884 (88%) isolates; many of the strains collected belonged to the *M. africanum* Afri_1 or *M. tuberculosis* sensu stricto lineages, and 86% of all *M. tuberculosis* strains were part of the EA lineage. Therefore, for the remainder of the analysis, we compared all *M. tuberculosis* sensu stricto isolates (including EA), EA lineage isolates separately, and *M. africanum* Afri_1 isolates. For the 2 major lineages (Afri_1 and EA), we identified 17–19 genotypes per 100 isolates, of which 9%–12% were found only once as singletons and thus were most likely the result of reactivation of previous disease (Table 1; Figure 1). The remaining spoligotypes (88%–91%) could be assigned to genotypic clusters with an average size of 11.8 and 13.3 isolates for *M. tuberculosis* EA and *M. africanum* Afri_1, respectively (Table 1). Assuming that recent transmission was correlated with cluster size and that each cluster contained 1 index case, the RTI_n–1_ for both populations indicated that 80%–83% of TB cases were attributable to newly acquired infections.

**Table 1:** Spoligotyping results for comparative population structure of *Mycobacterium tuberculosis* sensu stricto (including EA), the EA clade separately, and *M. africanum*, The Gambia, 2002–2009

Parameters	*M. tuberculosis* sensu stricto			*M. africanum*			Total
	All lineages	EA clade		Afri_1		Afri_2	
				With SIT 181	Without SIT 181		
Population parameters							
No. isolates (*n*)	548	467		334	136	2	884
No. genotypes	108	79		63	62	2	173
No. singletons (*s*)	60	43		41	41	2	103
No. clusters (*c*)	48	36		22	21	NA	70
Clustering rate, (*n* − *s*)/*n*	0.89	0.91		0.88	0.70	NA	0.88
Average cluster size, (*n* − *s*)/*c*	10.2	11.8		13.3	4.3	NA	11.2
Genetic diversity	40.0	27.0		22.7	43.4	NA	64.0
Recent transmission index	0.80	0.83		0.81	0.54	NA	0.80

*EA, Euro-American; SIT, shared international type; NA, not applicable.

Despite the similarities between the 2 mycobacterial populations, their individual compositions differed drastically. Whereas 59% of the *M. africanum* population was represented by only a single spoligotype, SIT 181, comprising 198 isolates, the same proportion of the *M. tuberculosis* EA population contained as many as 11–12 smaller clusters of comparable sizes; with SIT 53 being the largest cluster (Figure 1). Consequently, the θ variable, a maximum-likelihood estimate of the genetic diversity of a population (*11*), was lower for *M. africanum* (θ = 22.7) than for *M. tuberculosis* EA (θ = 27.0) or for all *M. tuberculosis* sensu stricto (θ = 43.3). The effect of SIT 181 on the population structure became especially apparent when this cluster was excluded from the analysis, resulting in multiple changes to *M. africanum* population parameters, such as clustering rate, cluster size, and genetic diversity θ (Table 1). The drop in RTI also demonstrates the contribution of SIT 181 to recent transmission within the *M. africanum* Afri_1 lineage.

### Spoligoforests of MTBC in West Africa

Using spoligoforests to display mycobacterial populations (*12*) takes the genetic relatedness of spoligotypes into consideration and allows deduction of relationships among bacterial sublineages. When we analyzed the 884 MTBC isolates from The Gambia, we found SIT 53 not only to be the most ancestral *M. tuberculosis* spoligotype but also to constitute the largest *M. tuberculosis* cluster (Technical Appendix Figure, panel A). Besides this ancestral strain, we identified 4 more recent major spoligotype clusters (SITs 42, 47, 50, and 61) and 5 third-generation clusters (SITs 20, 60, 62, 144, and 183). Most strains belonged to the modern *M. tuberculosis* lineages. Moreover, when the distribution and size of these individual clusters was considered, *M. tuberculosis* strains seemed to spread evenly within the host population, resulting in a uniformly distributed structure of the bacterial population. In contrast, the *M. africanum* population was highly skewed toward a central cluster of SIT 181, next to which (with the exception of SIT 187) no notable secondary spoligotype clusters emerged. Therefore, the population was concentrated around this spoligotype, and most cases of recently transmitted disease could be attributed to this genotype. Similar results were found when we reanalyzed a published spoligotype dataset from Guinea-Bissau (*6*), the West African country with the highest prevalence of *M. africanum* Afri_1 strains (Technical Appendix Figure, panel B).

### DESTUS Analysis

As indicated by cluster size analysis and RTI_n–1_, SIT 181 might be responsible for most recently transmitted TB cases in The Gambia. However, inferences about transmission that are purely based on cluster size analysis could be misleading because large clusters could equally be caused by an older strain that has been present for a long time or by strains that mutate slowly (*13*). To account for this imprecision, the DESTUS model was developed to factor the mutation rates of spoligotypes into analysis of genotypic clustering as a measure of emerging strains (*13*). DESTUS testing of our dataset for the whole MTBC or stratified by *M. tuberculosis* versus *M. africanum* found that SIT 181 was always detected as a highly significant emerging strain (p<10^−30^–10^−31^), followed by several other strains (Table 2). Similarly, SIT 181 was the only strain detected as emerging in the dataset from Guinea-Bissau (*6*).

**Table 2:** Emerging *Mycobacterium tuberculosis* complex strains as detected by DESTUS from samples collected in West Africa*

**Study site and years**	**p value†**
**Guinea Bissau, 1989–2008**	
SIT 181 (Afri_1)	3.8 × 10^–8^
SIT 187 (Afri_1)	4.7 × 10^–6^
**The Gambia, 2002–2009**	
SIT 181 (Afri_1)	2.5 × 10^–30^
SIT 187 (Afri_1)	5.1 × 10^–4^
** SIT 60 **(*M. tuberculosis*)	1.5 × 10^–6^
** SIT 61 (***M. tuberculosis*)	2.9× 10^–4^
** SIT 183 (***M. tuberculosis*)	3.9 × 10^–4^

*Years shown indicate when samples were collected. DESTUS, Detect Emerging Strains of Tuberculosis by Using Spoligotyping (*13*); SIT, shared international type; Afri_1, *M. africanum* West Africa 2. **†After Sidak Dunn correction for type I error.**

### Detecting Temporal Clusters of *M. africanum* SIT 181

To confirm previous results and to detect high rates of temporal clustering, we applied a purely temporal analysis to the *M. africanum* population by using the discrete Poisson model in SaTScan version 9.1.1 (www.satscan.org) (Table 3). We identified a significant (p = 0.001) temporal cluster of SIT 181 cases during August 2007–June 2008. This cluster showed an increased relative risk of 2.65 to the population for contracting SIT 181 when compared with the risk across the full study period (2002–2009).

**Table 3:** Significant temporal clustering of human cases of infection with *Mycobacterium africanum* SIT 181, The Gambia, August 14, 2007–June 3, 2008*

No. cases		Relative risk	LLR	p value
Actual	Expected			
48	21.30	2.65	14.424230	0.001

*Detected with SaTScan version 9.1.1 software (www.satscan.org) (*14*) by applying retrospective purely temporal analysis using the discrete Poisson model. SIT, shared international type; LLR, log-likelihood ratio.

### Immunogenicity of MTBC Isolates

To identify associations between the emergence of certain genotypes and their immunogenicity, we used ELISPOT to measure PPD-induced interferon-γ responses in blood samples collected during 2002–2007 from patients infected with *M. tuberculosis* sensu stricto (n = 235), *M. tuberculosis* EA (n = 194), and *M. africanum* (n = 137). On the basis of the assumption that clustering is indicative of recent transmission, we compared PPD ELISPOT positivity of clustered strains with singletons (Table 4). Our data suggest that *M. africanum* strains clustered by spoligotyping are significantly more likely to produce a positive PPD ELISPOT result than are singletons (OR 31.78, 95% CI 9.24–109.28; p = 0.0001). For *M. tuberculosis* sensu stricto and *M. tuberculosis* EA, we found a similar, yet not significant, tendency.

**Table 4:** PPD ELISPOT results for blood samples from patients infected with *Mycobacterium tuberculosis* sensu stricto (including EA lineage), *M. tuberculosis* EA lineage, and *M. africanum* strains, The Gambia, 2002–2007*

Strain and result	No. spoligotypes	Odds ratio (95% CI)	p value

Clustered	Singleton	Total	χ^2^ test†	Fisher exact test‡
*M. tuberculosis*						
Positive	182	19	201	1.65 (0.57–4.77)	0.3495	0.2533
Negative	29	5	34	0.61 (0.21–1.75)	0.3495	0.2533
Total	211	24	235			
*M. tuberculosis* EA						
Positive	149	14	163	1.58 (0.48–5.15)	0.4479	0.3184
Negative	27	4	31	0.63 (0.19–2.07)	0.4479	0.3184
Total	176	18	194			
*M. africanum*						
Positive	110	9	119	31.78 (9.24–109.28)	0.0001	0.0001
Negative	5	13	18	0.03 (0.01–0.11)	0.0001	0.0001
Total	115	22	137			
*M. africanum*	SIT 181					
Positive	73	9	82	21.09 (6.09–73.04)	0.0001	0.0001
Negative	5	13	18	0.05 (0.01–0.16)	0.0001	0.0001
Total	78	22	100			

*PPD, purified protein derivative; EA, Euro-American. †2-tailed. ‡1-tailed.

After applying more stringent criteria than mere clustering, such as determining emerging strains (DESTUS), we compared PPD ELISPOT positivity of SIT 181 to singleton *M. africanum* Afri_1 strains; this analysis confirmed that SIT 181 is significantly more immunogenic than other types (OR 21.09, 95% CI 6.09–73.04; p = 0.0001). Similarly, we found a slight, not significant tendency for patients infected with SIT 181 to be more likely than patients infected with singleton strains to yield a positive Mantoux skin test (OR 1.15, 95% CI 0.30–4.44).

## Discussion

We found a correlation between MTBC strains of higher immunogenicity and their ability to spread within the human host population. In the study population in The Gambia, patients infected with strain SIT 181, the most prevalent *M. africanum* strain, were significantly more likely to yield a positive PPD ELISPOT result than were patients infected with *M. africanum* strains that do not have the ability to establish themselves within the human host population. To describe this association, we constructed a detailed population structure of MTBC isolates in which we analyzed 884 spoligotypes obtained from mycobacterial isolates from TB cases identified during 2002–2009. We found that most circulating strains belonged to either the EA or *M. africanum* Afri_1 clades, and therefore, we specifically focused on the comparison between these 2 lineages. On the basis of the assumption that clustered strains indicate recent transmission, we found that several *M. tuberculosis* strains appeared to have spread evenly within the host population. In contrast, most (59%) *M. africanum* transmission events and infections could be attributed to spoligotype SIT 181, which was responsible for 22% of all TB cases in the country. This result not only confirms recent findings from Guinea-Bissau, in which SIT 181 caused up to 49% of all *M. africanum* infections (*6*), but is also in agreement with a previous study of a smaller set of isolates from this study that showed a similar strain distribution within the 2 major lineages (Afri_1 and EA) (*17*).

Although it is widely accepted that genetic clusters are indicative of recent transmission, caution must be taken with the interpretation of such findings because the successful spread of a strain within the population—and thus genetic clustering—is highly dependent on 2 properties of the bacteria. For successful spread, strains must transmit from infected to uninfected host first; after this initial transmission, the infection must progress to active disease to be transmitted to the next susceptible host. However, only case–contact tracing studies, not molecular clustering data alone, can distinguish between transmission and progression of the different lineages. Consequently, we refer here to the spreading capacity of strains, rather than to transmission.

Our analysis using DESTUS (*13*), which correctly predicted the widely accepted emergence of Beijing lineage *M. tuberculosis* strains in other studies (*13*), detected SIT 181 as an unusually fast-growing strain relative to the mycobacterial background population of the sample. However, DESTUS does not take into account the migration history of Europeans and the mycobacteria they introduced into Africa. Because of this limitation, we sought to confirm our finding with a second approach and conducted a purely temporal analysis. We found that SIT 181 could have been emerging during a certain time in the study period, identifying a temporal cluster during 2007–2008 for which risk for infection with SIT 181 was 2.65-fold higher than that for the whole study period (2002–2009).

With SIT 181 constituting such a prominent cluster, it is conceivable that the strain’s high prevalence is related to selective pressure through, for instance, antimicrobial drug therapy. However, because resistance rates are relatively low in The Gambia (*18*), this explanation does not seem to apply. Therefore, we suggest another selective mechanism: we believe differences in spreading capacity and the interaction between *M. tuberculosis* and *M. africanum* populations might play a crucial role. In contrast to *M. africanum*, several clustered strains within the *M. tuberculosis* population have comparable potential to spread within the human host population, but no strain has a notable advantage over another, which results in a well-balanced population structure. We hypothesize that SIT 181, in its expanded ability to spread, resembles these *M. tuberculosis* strains more than it does strains with other spoligotype patterns in the *M. africanum* lineage. Thus, SIT 181 has a selective advantage and is able to compete with *M. tuberculosis* for the same biological niche within the human host.

**Figure 2:**
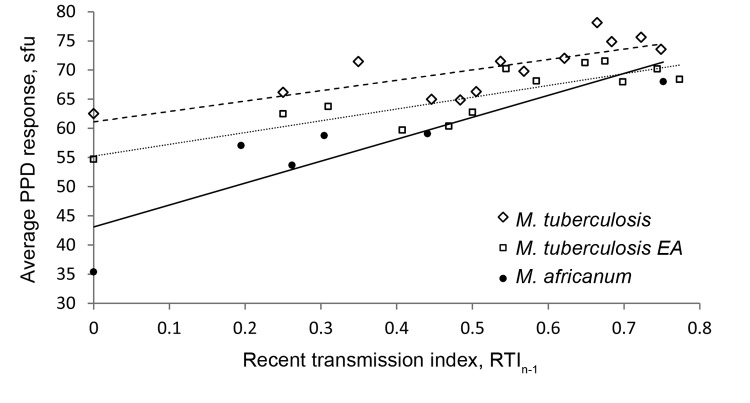
Linear regression analysis showing correlation between average quantitative purified protein derivative (PPD) response and recent transmission index (RTI_n−1_) for *Mycobacterium tuberculosis* complex isolates, The Gambia, 2002–2007. Open diamonds and dashed line, *M. tuberculosis* sensu stricto, including Euro-American (EA) lineage (R^2^ = 0.606); open squares and dotted line, *M. tuberculosis* EA lineage (R^2^ = 0.7272); black circles and solid line, *M. africanum* Afri_1 lineage (R^2^ = 0.7732). sfu, spot-forming units.

The nature and extent of epitope variation in *M. tuberculosis* strains is unclear. Findings range from highly variable T-cell epitopes within the *esx* gene family (*19*) to highly conserved epitopes when comparing genetic variation between predicted epitopes and the remainder of the *M. tuberculosis* genome (*7*). The latter study, which described T-cell epitopes as highly conserved, suggests that hyperconservation of T-cell epitopes is beneficial for the bacteria and could result in the successful spread of strains. In our study, we sought to understand whether the magnitude of an induced immune response was correlated with the spreading capacity of the bacteria, with a special focus on SIT 181. We therefore investigated PPD ELISPOT responses of patients infected with either lineage and found a nonsignificant tendency for clustered *M. tuberculosis* sensu stricto and EA strains toward being more likely than singletons to produce a positive PPD result. This small difference in immunogenicity might be in line with our hypothesis that the *M. tuberculosis* population spreads fairly homogenously. Consistent with the large observed differences in spreading capability, SIT 181 or clustered *M. africanum* strains have a 20- to 30-fold higher probability of yielding a positive PPD response (p<0.0001). This positive correlation between immunogenicity and spread becomes even more apparent when the RTI is plotted against the average quantitative PPD response (Figure 2). This comparison demonstrates not only the expected larger range in immunogenicity within the *M. africanum* lineage but also the phenotypic relatedness in immunogenicity of the highly spreading strains, independent of lineage.

An association between PPD response and spreading capacity is conceivable. A previous publication found that household contacts who slept in the same bedroom as an index case-patient (i.e., who were exposed to the highest infectious loads) had higher PPD ELISPOT responses than did less-exposed household contacts (*20*). However, further analysis will be needed to conclusively address whether spreading capacity is determined by infectious load; smear-positivity grade of cases; magnitude of the induced immune response; or an as-yet unknown immunogenic protein that enhances transmissibility, the absence of which (from strains of low spread) is merely reflected by a reduced ELISPOT response to PPD.

One possible limitation of spoligotype data is that the technique was designed on the basis of the clustered regularly interspaced short palindromic repeats (CRISPR) regions of *M. tuberculosis* sensu stricto and, therefore, could have a lower resolution when applied to *M. africanum*. Consequently, the large observed SIT 181 cluster could be a result of misclassification. Although the CRISPR regions of *M. tuberculosis* and *M. africanum* have not been extensively compared, we believe misclassification is very unlikely for 2 reasons. First, we found a comparable resolution of the technique for both lineages (18–19 genotypes/100 isolates). Second, when calculating the Hunter-Gaston Index (HGI), a measure for the discriminatory power of a technique, we found that HGI = 0.96 for spoligotyping of *M. tuberculosis*, HGI = 0.94 for *M. africanum* excluding SIT 181, and HGI = 0.64 for *M. africanum* with SIT181. Spoligotyping works equally well for 41% of *M. africanum* isolates and for *M. tuberculosis* isolates (0.94 vs. 0.96) but has drastically worse discriminatory power for the remaining 59% of *M. africanum* strains (0.64). A drop in HGI that was a result of misclassification within the *M. africanum* lineage could only result from a CRISPR region or mutation rate that was notably different between SIT 181 and the other *M. africanum* strains. However, this is unlikely because the remaining strains with HGI = 0.94 evolved out of SIT 181 and, thus, most likely have identical CRISPR regions and mutation rates. Therefore, by comparing these 3 HGI results, we can conclude that SIT 181 is a real cluster and not a result of misclassification. High-resolution genotyping methods, such as mycobacterial interspersed repetitive unit–variable number tandem repeat typing or whole-genome sequencing, is needed to conclusively confirm the genotypic homogeneity of the group of strains that constitute spoligotype pattern SIT 181.

We conclude that spoligotyping possesses comparable discriminatory resolution for *M. tuberculosis* and *M. africanum*. We were able to demonstrate that SIT 181 represents a strain (or family of strains) that clusters genotypically, temporally, and phenotypically and represents a major public health concern in West Africa, responsible for nearly one fourth of TB cases in The Gambia (22%) and Guinea-Bissau (23%) (*6*). Deciphering the virulence mechanisms that determine the differences in immunogenicity and spreading capacity between SIT 181 and the remaining singleton *M. africanum* strains will be key to improving TB prevention and transmission control in this region.

Technical Appendix Spoligoforests showing relationships between *Mycobacterium tuberculosis* complex isolates for 884 isolates from The Gambia, collected during 2002–2009, and 414 isolates from Guinea-Bissau, collected during 1989–2008.
