# Supplementary material for: Immunogenic Mycobacterium africanum Strains Associated with Ongoing Transmission in The Gambia
Source: Emerg Infect Dis. 2013 Oct;19(10):1593–604. doi: 10.3201/eid1910.121023 (PMC3810728; doi:10.3201/eid1910.121023)
Supplement: Technical Appendix — Spoligoforests showing relationships between Mycobacterium tuberculosis complex isolates for 884 isolates from The Gambia, collected during 2002–2009, and 414 isolates from Guinea-Bissau, collected during 1989–2008. [file 12-1023-Techapp-s1.pdf]

Article DOI: http://dx.doi.org/10.3201/eid1910.121023

Immunogenic *Mycobacterium africanum* Strains Associated with Ongoing Transmission in The Gambia

Technical Appendix

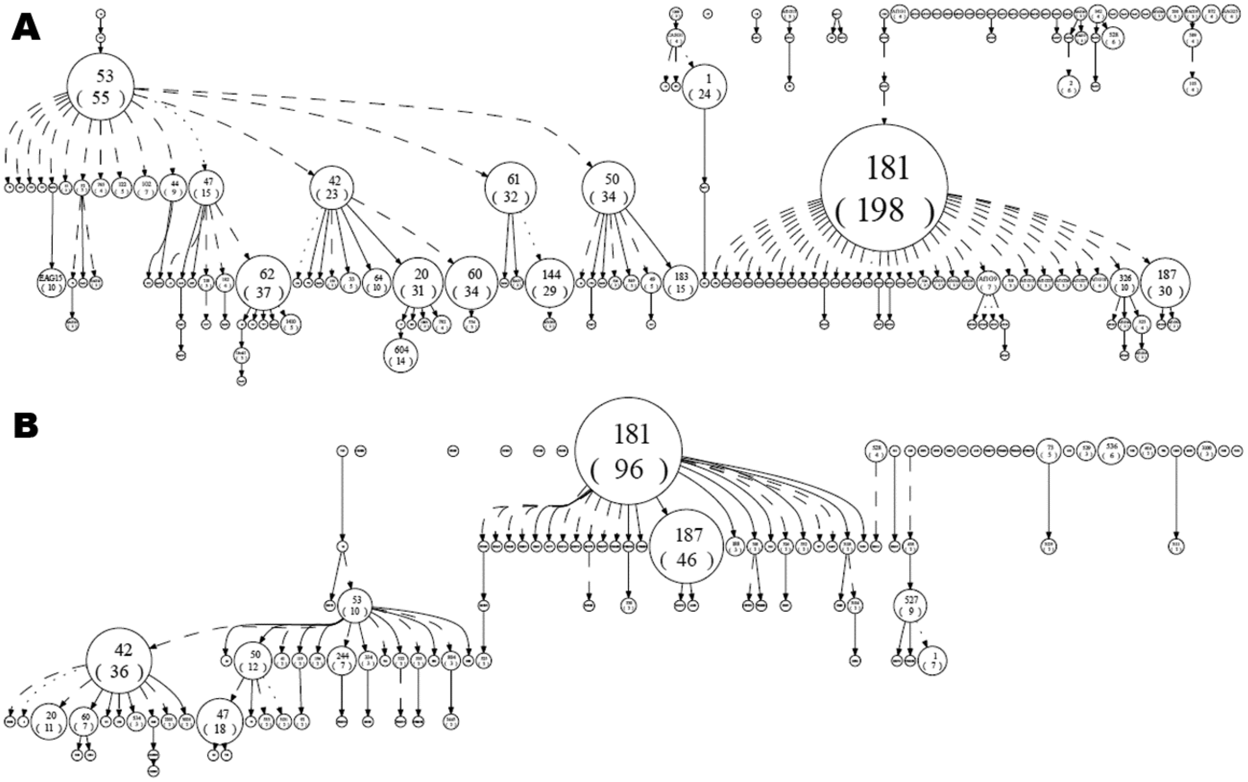

Technical Appendix Figure. Spoligoforests showing relationships between *Mycobacterium tuberculosis* complex isolates for A) 884 isolates from The Gambia, collected during 2002–2009, and B) 414 isolates from Guinea-Bissau, collected during 1989–2008 (*1*). Spoligoforests were created by using spolTools (www.emi.unsw.edu.au/spolTools). Numbers within each node indicates the shared international type number assigned to each spoligotype by the SpolDB4 database; numbers in parentheses indicate cluster size.

Reference

1. Groenheit R, Ghebremichael S, Svensson J, Rabna P, Colombatti R, Riccardi F, et al. The Guinea-Bissau family of *Mycobacterium tuberculosis* complex revisited. PLoS ONE. 2011;6:e18601. PubMed http://dx.doi.org/10.1371/journal.pone.0018601
